# A Case of Bundle Branch Re-entrant Ventricular Tachycardia 1 Year After Transcatheter Aortic Valve Replacement

**DOI:** 10.19102/icrm.2022.130804

**Published:** 2022-08-15

**Authors:** Amarbir Bhullar, Nikhil Sharma, Rosaline Ma, Tia Bimal, Umair Ansari, Stavros Mountantonakis

**Affiliations:** ^1^Department of Cardiology, Zucker School of Medicine at Hofstra/Northwell, Lenox Hill Hospital, New York, NY, USA; ^2^Department of Medicine, Zucker School of Medicine at Hofstra/Northwell, Lenox Hill Hospital, New York, NY, USA; ^3^Department of Medicine, Zucker School of Medicine at Hofstra/Northwell, Mather Hospital, Port Jefferson, New York, NY, USA

**Keywords:** Bundle branch re-entrant ventricular tachycardia, macro–re-entrant, syncope, TAVR

## Abstract

Bundle branch re-entrant ventricular tachycardia (VT) (BBR-VT) is a unique type of ventricular tachycardia often seen in patients with advanced heart diseases. Rarely, it is found in patients with a structurally normal heart. We describe a case of BBR-VT in a patient with normal ventricular function, a year after transcatheter aortic valve replacement (TAVR) for aortic stenosis. A 73-year-old man with a past medical history of non-obstructive coronary artery disease and severe aortic stenosis status post-TAVR with a 23-mm Sapien valve (Edwards Lifesciences, Irvine, CA, USA) about 1 year prior presented with palpitations and syncope. The electrocardiogram (ECG) showed a wide complex tachycardia with a left bundle branch block (LBBB) pattern and atrioventricular dissociation. The tachycardia was incessant and paroxysmal during 24-h telemetry monitoring. An electrophysiology study showed a normal A–H interval of 90 ms and a prolonged H–V interval of 84 ms with evidence of a split His. A hemodynamically stable VT was induced with a cycle length of 453 ms, which was identical to the clinical VT. This was diagnosed to be BBR-VT given the typical ECG pattern of LBBB, the presence of His inscription before each ventricular signal, and the H–H interval variation–predicted V–V variation when there was a wobble in tachycardia cycle length. Injury of the His–Purkinje system post-TAVR can provide the substrate for the development of BBR-VT. Current published literature shows early occurrence post-TAVR, but our case suggests that the timing between the index procedure and arrhythmia occurrence can be variable.

## Introduction

Bundle branch re-entrant ventricular tachycardia (BBR-VT) is a unique type of ventricular tachycardia (VT) that involves the right and left bundle branches and the ventricular septum as components of the macro–re-entrant circuit. BBR-VT is often seen in patients with advanced heart diseases, such as dilated cardiomyopathy, coronary artery disease, valvular heart disease, and myotonic dystrophy^1–7^ with concomitant severe His–Purkinje system (HPS) disease. Occasionally, it can be seen in patients with a structurally normal heart as the unique electrophysiological properties of rapid conduction and long refractory periods ordinarily prevent sustained re-entry within the HPS. We describe a case of BBR-VT in a patient with normal ventricular function, 11 months after transcatheter aortic valve replacement (TAVR) for aortic stenosis.

## Case Presentation

**Figure 1:**
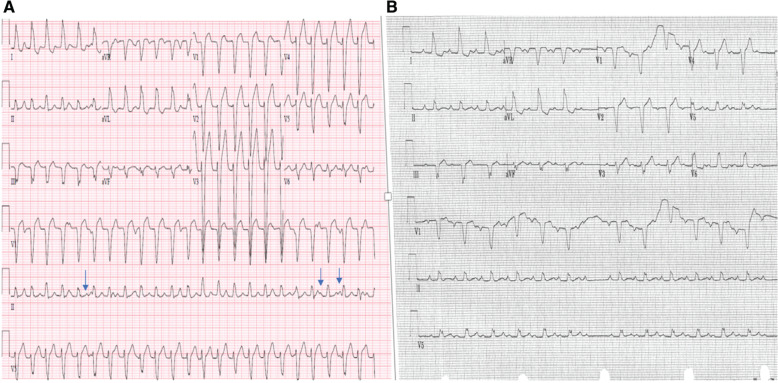
**A:** Wide complex tachycardia with a left bundle branch block morphology at a rate of 150 bpm. Atrioventricular dissociation and electrical alternans are seen. **B:** Baseline 12-lead electrocardiogram.

**Figure 2:**
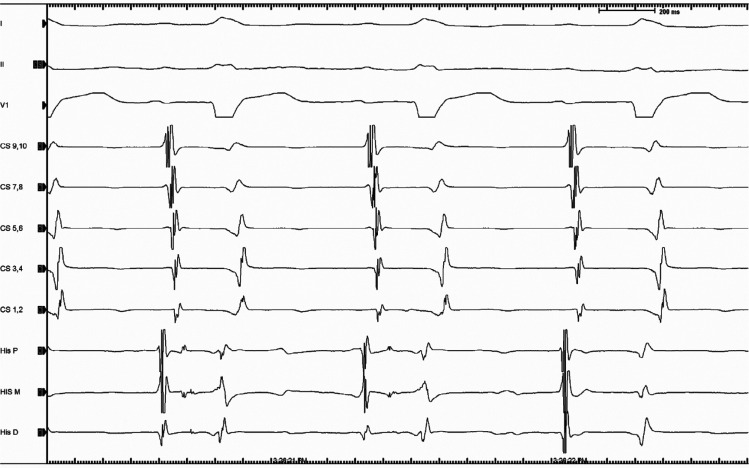
Split His seen on His catheter.

**Figure 3:**
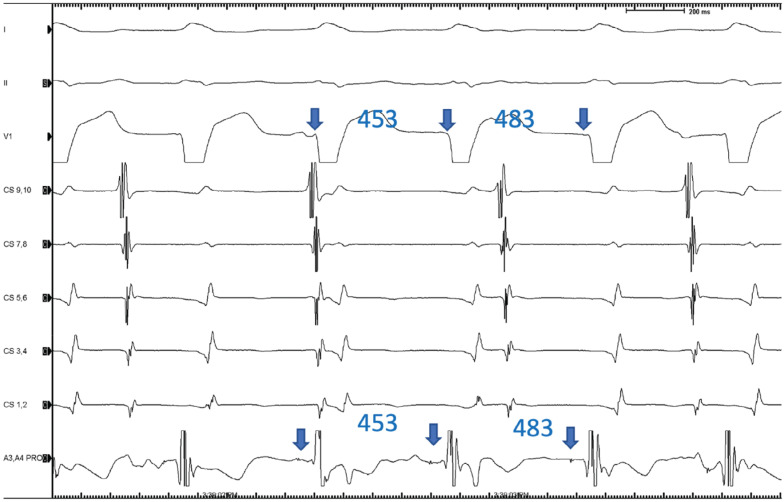
His inscription before each ventricular signal and H–H interval variation predicting V–V variation during a wobble in tachycardia cycle length.

A 73-year-old man with a past medical history of non-obstructive coronary artery disease, with preserved ventricular function and severe aortic stenosis status post-TAVR with a 23-mm Sapien valve (Edwards Lifesciences, Irvine, CA, USA) 11 months prior, presented with palpitations and syncope. A presenting 12-lead electrocardiogram (ECG) showed a wide complex tachycardia at a rate of 150 bpm with a left bundle branch block (LBBB) pattern, a QRS width of 135 ms, and a left inferior axis with a precordial transition to positive at lead V5. Electrical alternans was seen. Dissociation was noted between the QRS complexes and P-waves, supporting the diagnosis of VT **(Figure 1A)**. Baseline 12-lead ECG showed a sinus rhythm with first-degree atrioventricular (AV) block and LBBB with a similar but not identical morphology with the QRS complex while in tachycardia. Of note, LBBB had developed after the TAVR procedure. While in sinus rhythm, there were non-conducting P-waves without change in the R–R or P–R interval, suggesting type 2, second-degree AV block **(Figure 1B)**. The tachycardia was paroxysmal during a 24-h telemetry monitoring. An electrophysiology (EP) study was performed, which showed a normal A–H interval of 90 ms and a prolonged H–V interval of 84 ms with evidence of a split His **(Figure 2)**. Programmed ventricular stimulation demonstrated the absence of retrograde conduction at baseline and on isoproterenol. A hemodynamically stable VT was induced with burst ventricular pacing. The lack of a proper His signal limited our ability to identify any retrograde V–H prolongation at the time of tachycardia initiation. The tachycardia had a cycle length of 453 ms and was identical in morphology to the clinical VT. When induced, the VT was slower in rate compared to the presenting tachycardia and secondary to the effects of sedation. This was diagnosed to be BBR-VT based on the typical ECG pattern of LBBB, the presence of His inscription before each ventricular signal, and the H–H interval variation-predicted V–V variation when there was a wobble in tachycardia cycle length **(Figure 3)**. The tachycardia was not sustained enough to perform entrainment maneuvers. Based on the presence of severe HPS disease and the behavior of BBR-VT, a decision was made to proceed with radiofrequency ablation of the apical most inscription of the right bundle potential. Ideally, the left bundle should have been mapped but was not in this case as we felt that the patient would eventually need a pacemaker. Ablation was performed using a 4-mm non-irrigated tip catheter, which resulted in immediate termination of the tachycardia with concomitant complete heart block. The patient subsequently underwent dual-chamber permanent pacemaker implantation.

## Discussion

BBR-VT is a unique macro–re-entrant VT that involves the right bundle, left bundle, and ventricular septum as part of the circuit. Akhtar et al. first documented re-entry within the HPS in humans in studies involving isolated ventricular beats commonly produced by programmed ventricular stimulation (V_3_ phenomenon).^8,9^ These isolated re-entrant beats within the HPS represent a normal response to stimulation. It is well recognized that sustained bundle branch re-entry cannot be induced in patients with a normal HPS. The latter may be explained by the electrophysiological properties of a normal HPS characterized by the combination of very fast conduction velocity and a relatively long refractory period which precludes the formation of a stable re-entry circuit.^10^ However, in conditions when the conduction in the HPS is prolonged because of disease or drugs, sustained re-entry within the bundle branches is facilitated. It accounts for 6% of inducible sustained monomorphic VT during EP studies.^11^ A wide QRS tachycardia with an LBBB morphology is the most common form of BBR-VT encountered in clinical practice, in which the right bundle branch (RBB) serves as the antegrade limb, the left bundle serves as the retrograde limb, and the interventricular septum provides the connecting link. BBR-VT with an RBB configuration, where the reverse sequence of activation occurs, is less commonly encountered.^12^ It is often seen in patients with acquired heart diseases, which involve the HPS, leading to slow conduction within the Purkinje system and facilitating the occurrence of BBR-VT. Rarely, it can be seen in patients without structural heart disease: Li et al.^13^ found that prolonged H–V interval in sinus rhythm is not a prerequisite for BBR-VT. In their study of 13 patients with BBR-VT, 6 out of 13 patients had normal H–V intervals in sinus rhythm; however, all 6 patients had functional His–Purkinje abnormalities. Rare case reports of BBR-VT following surgical aortic valve replacement and TAVR have been published.^12,14–17^ Our literature search showed 4 case reports of BBR-VT following TAVR and all of them occurred in the first 2 weeks post-TAVR. A prolonged H–V interval was reported in 3 cases and 1 case did not report the H–V interval. The unique feature of our case is that the patient presented with BBR-VT 11 months following TAVR, suggesting that factors including valve expansion, cardiac remodeling, and progression of injury to HPS could influence the timing of BBR-VT occurrence. TAVR has become a well-established percutaneous procedure for patients with symptomatic severe aortic stenosis; however, it is often complicated by conduction abnormalities and the need for permanent pacemaker implantation in 8%–12.5% of cases post-TAVR.^18^ The role for an EP study post-TAVR has not yet been well defined, but studies have shown worsening of conduction parameters in up to 78% of patients undergoing CoreValve (Medtronic, Minneapolis, MN, USA) implantation.^19^ The long-term effects of this injury to HPS are unclear; however, there are disturbing reports of sudden cardiac death at 1 year post-TAVR with an incidence of 1%–2.5%.^20,21^ The mode of sudden cardiac death for this patient population is unclear. Nevertheless, ventricular arrhythmias of unclear mechanism have also been reported.^22^ Our case highlights and adds to the existing literature on the occurrence of BBR-VT based on new conduction abnormalities and particularly HPS disease post-TAVR.

## Conclusion

Injury of the HPS post-TAVR can provide the substrate for the development of BBR-VT. Our case adds to the limited number of BBR-VT cases published in the literature and suggests that the timing between the index procedure and arrhythmia occurrence can vary.
